# A multicenter prospective clinical trial reveals cell-free DNA methylation markers for early esophageal cancer

**DOI:** 10.1172/JCI186816

**Published:** 2025-02-25

**Authors:** Ruixiang Zhang, Yongzhan Nie, Xiaobing Chen, Tao Jiang, Jinhai Wang, Yuhui Peng, Guangpeng Zhou, Yong Li, Lina Zhao, Beibei Chen, Yunfeng Ni, Yan Cheng, Yiwei Xu, Zhenyu Zhu, Xianchun Gao, Zhen Wu, Tianbao Li, Jie Zhao, Cantong Liu, Gang Zhao, Jiakuan Chen, Jing Zhao, Gang Ji, Xiaoliang Han, Jie He, Yin Li

**Affiliations:** 1 Department of Thoracic Surgery, National Cancer Center/National Clinical Research Center for Cancer/Cancer Hospital, Chinese Academy of Medical Sciences and Peking Union Medical College, Beijing, China.; 2 State Key Laboratory of Holistic Integrative Management of Gastrointestinal Cancers, National Clinical Research Center for Digestive Diseases, Xijing Hospital, Fourth Military Medical University, Xi’an, Shaanxi, China.; 3 Department of Oncology, Affiliated Cancer Hospital of Zhengzhou University, Henan Cancer Hospital, Zhengzhou, Henan, China.; 4 Department of Thoracic Surgery, Tangdu Hospital, The Air Force Military Medical University, Xi’an, Shaanxi, China.; 5 Department of Gastroenterology, Second Affiliated Hospital, Xi’an Jiaotong University, Xi’an, Shaanxi, China.; 6 Department of Clinical Laboratory Medicine, Cancer Hospital of Shantou University Medical College, Shantou, Guangdong, China.; 7 BioChain (Beijing) Science & Technology Inc., Beijing, China.; 8 Department of Radiation Oncology and; 9 Department of Digestive Surgery, Xijing Hospital, Fourth Military Medical University, Xi’an, Shaanxi, China.

**Keywords:** Clinical trials, Oncology, Cancer, Molecular diagnosis

## Abstract

**BACKGROUND:**

Current methods for detecting esophageal cancer (EC) are generally invasive or exhibit limited sensitivity and specificity, especially for the identification of early-stage tumors.

**METHODS:**

We identified potential methylated DNA markers (MDMs) from multiple genomic regions in a discovery cohort, and a diagnostic model was developed and verified in a model-verification cohort of 297 participants. The accuracy of the MDM panel was validated in a multicenter, prospective cohort (*n* = 1,429). The clinical performance of identified MDMs were compared with current tumor-associated protein markers.

**RESULTS:**

From 31 significant differentially methylated EC-associated regions identified in the marker discovery, we trained and validated a 3-MDM diagnostic model that could discriminate among patients with EC and volunteers without EC in a multicenter clinical prospective cohort with a sensitivity of 85.5% and a specificity of 95.3%. This panel showed higher sensitivity in diagnosing early-stage tumors, with sensitivities of 56% for stage 0 and 77% for stage I, compared with the performance of current biochemical markers. In population with high risk for EC, the sensitivity and specificity were 85.68% and 93.61%, respectively.

**CONCLUSION:**

The assessment of tumor-associated methylation status in blood samples can facilitate noninvasive and reliable diagnosis of early-stage EC, which warrants further development to expand screening and reduce mortality rates.

**TRIAL REGISTRATION:**

ChiCTR2400083525.

**FUNDING:**

Science and technology funds of Beijing Municipal Science & Technology Commission, Administrative Commission of Zhongguancun Science Park. Project number: Z201100005420007.

## Abstract 2

A blood-testing method based on DNA methylation markers shows excellent performance in detecting early-stage esophageal cancer, improving early diagnosis and potentially reducing mortality rates.

## Introduction

Esophageal squamous cell carcinoma (ESCC) remains the predominant histological subtype of esophageal cancer (EC) worldwide, representing nearly 90% of the 604,000 new cases reported in 2020 (1). As a result of the absence of common clinical manifestations and physical indicators during the initial phases of EC, a large proportion of patients in China are diagnosed at intermediate-to-advanced stages, which contributes to the unfavorable overall prognosis for individuals with this condition (2). However, regions in China where screening is routine demonstrate notably higher survival rates compared with those without screening (40.6% vs 32.8%) (3). In addition, substantial improvements in survival are observed when the disease is confined to superficial mucosal layers, with rates exceeding 80% after endoscopic or surgical intervention (4), suggesting the critical value of early detection in clinical practice. However, endoscopy, the gold-standard technique for EC diagnosis, is not suitable for population-based screening owing to its relatively high cost and invasiveness. In addition, although the preinvasive stage of esophageal squamous dysplasia is well-described and could serve as a reliable basis for development of less invasive, blood-based, early-detection strategies, currently available biomarkers have shown generally insufficient accuracy and efficacy (5).

To overcome the deficiency, cancer-specific DNA methylation modifications have been proposed as potentially promising biomarkers for EC detection owing to their prominent role in dysregulation of tumor suppressor genes or oncogenes and, consequently, tumorigenesis (6). Aberrant methylated DNA markers (MDMs) have been demonstrated to appear early in oncogenesis and presented less heterogeneously than gene mutations; thus, they could serve as ideal tools for early detection of different malignancy types, including EC. For example, MDMs identified from esophageal cytology specimens obtained via sponge sampling devices showed nearly perfect performance in detecting Barrett’s esophagus, achieving 92% sensitivity and 94% specificity (7). This high accuracy suggests that MDMs could provide high diagnostic power for early detection of EC. Another study has shown that a panel of 5 MDMs (FER1L4, ZNF671, ST8SIA1, TBX15, and ARHGEF4) identified from tissue samples could also to detect EC in plasma-based assays from limited clinical samples (8). It is necessary to prove that at an early stage, such as at stage 0, EC can be detected in plasma and to validate the early diagnosis of EC in a clinical trial with sufficient clinical samples, including EC at stage 0.

To improve early detection of EC, in this current study, we developed a method for detecting MDMs in multiple genomic regions in EC blood samples at early stage. We applied this method in a training cohort, which resulted in a diagnostic model based on 3 markers for EC at early stage. Then, we validated the model in a multicenter clinical cohort, including a diagnosed group and diagnosing group with high risk of EC to simulate applications of methylation testing in real clinical situation. Furthermore, we systematically validated the diagnostic performance of these EC-specific methylation markers for detecting early-stage EC by comparison with current protein-based markers.

## Results

**Figure 1:**
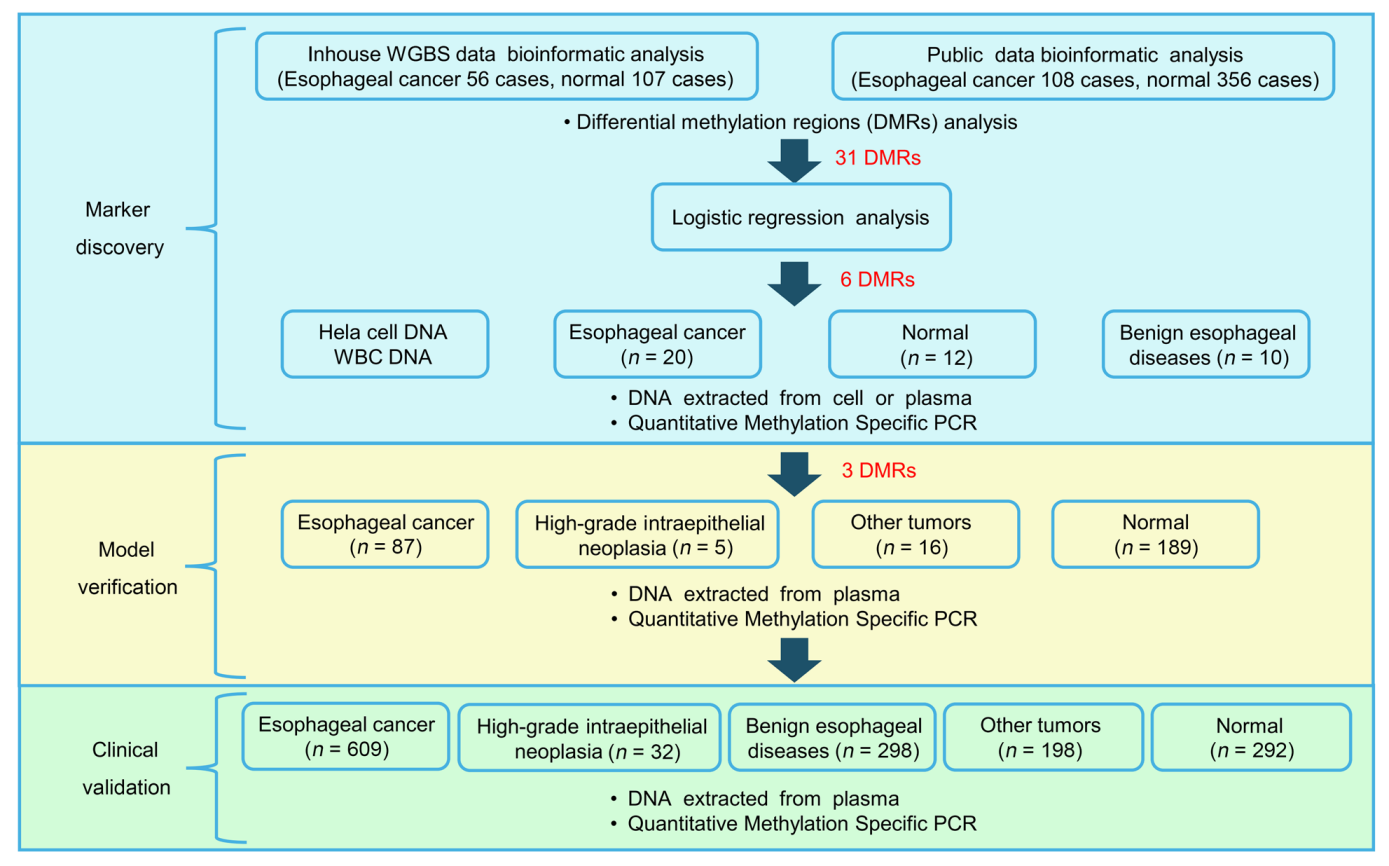
Workflow of the 3 stages of study design, including marker discovery, model verification, and clinical validation.

The study was conducted in 3 main phases: (a) marker discovery, a phase in which methylation markers were screened from tissue and plasma samples; (b) model verification, a phase in which the probes were optimized, and the model was constructed; and (c) clinical validation, a phase wherein diagnostic performance for early EC was evaluated in a cohort of 1,429 participants and individuals acting as controls (Figure 1).

### Marker discovery

**Figure 2:**
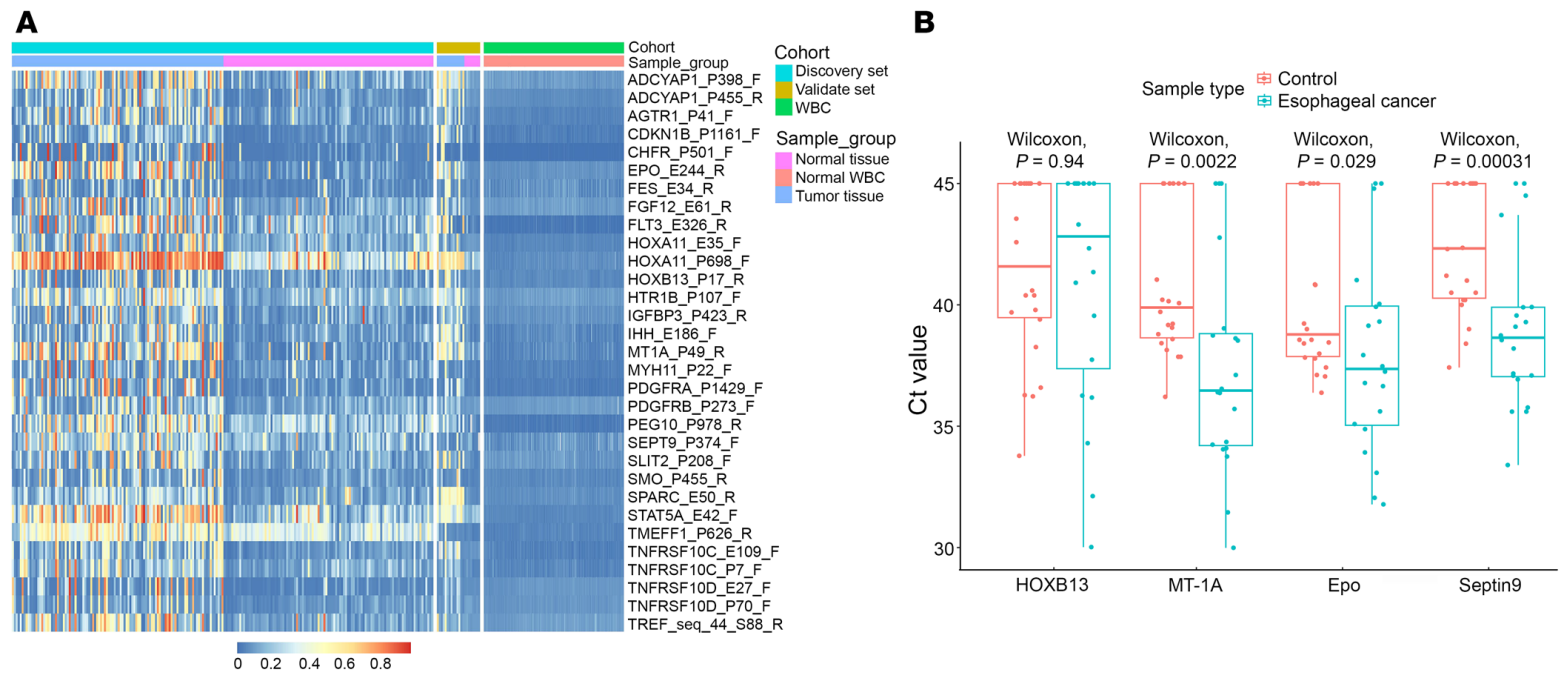
Differential methylation of candidate DNA markers between patients with esophageal cancer and and healthy individuals. (**A**) Methylation levels of 31 differentially methylated regions between ESCC tumor tissue (*n* = 108) and nonesophageal cancer cases (normal tissue and WBC) (*n* = 356) derived from public datasets, illustrating distinct methylation profiles between cancerous and noncancerous samples. (**B**) Box plots presenting the qMSP Ct values for the selected 4 potential markers in blood samples of patients with esophageal cancer (*n* = 20) and control cases (*n* = 22) in the marker discovery cohort. Control cases included individuals with benign esophageal diseases and and healthy individuals. The box-and-whisker plots illustrate the IQR, with the line within the box denoting the median of the data and the whiskers extending from the box to the minimum and maximum values within 1.5 times the IQR. Each point represents 1 sample. Wilcoxon’s test was used for pairwise comparison.

In the biomarker discovery phase, we analyzed whole-genome bisulfite sequencing (WGBS) data from plasma samples of 56 patients with EC and 107 healthy individuals acting as controls. This dataset was augmented with the methylation data from 108 ESCC samples and 356 samples from healthy individuals sourced from a public database (GEO GSE51287, GSE26784, GSE40279, GSE52826, and GSE74693) to delineate differential methylation patterns. Our analysis revealed that overall methylation predominated in relatively rare genomic regions (Supplemental Figure 1; supplemental material available online with this article; https://doi.org/10.1172/JCI186816DS1). We identified 31 differentially methylated regions associated with EC, and some of the selected genes were shown in Integrative Genomics Viewer (Figure 2A and Supplemental Figure 2). Using logistic regression modeling, we preliminarily identified 6 differentially methylated regions, which were annotated to the 6 genes, Epo, MT-1A, PDGFRA, HOXB13, TRIM15, and Septin9, as potential diagnostic markers for EC. These 6 potential diagnostic markers were further validated using quantitative methylation-specific PCR (qMSP) in Hela cell DNA, white blood cell (WBC) DNA, and plasma samples collected from a subset of participants, including 20 patients with EC, 12 healthy individuals, and 10 patients with benign esophageal diseases. The original data of qMSP assays based on these plasma samples were presented in Supplemental Table 1.The methylation level of the differentially methylated region on the TRIM15 gene showed relatively high background signal in WBC DNA, and the amplification signal of the differentially methylated region on the PDGFRA gene in Hela cell DNA was below the limit of detection, which led us to focus on the remaining 4 potential markers (Epo, MT-1A, HOXB13, and Septin9) for further verification (Supplemental Figure 3). Notably, the Ct values in qMSP assays showed that the differentially methylated regions on Septin9, MT-1A, and Epo genes were significantly lower in EC samples compared with controls (Wilcoxon’s test, *P* < 0.05), indicating these potential markers had a higher methylation status in cancer samples (Figure 2B). Through this validation process, 3 highly unique differentially methylated regions emerged as candidate MDMs.

### Model verification

In the model verification phase, the 3 candidate MDMs were assessed by qMSP in plasma samples of the model verification cohort, including 87 patients with EC, 5 patients with high-grade intraepithelial neoplasia, 16 patients with other types of cancers, and 189 healthy individuals.

**Figure 3:**
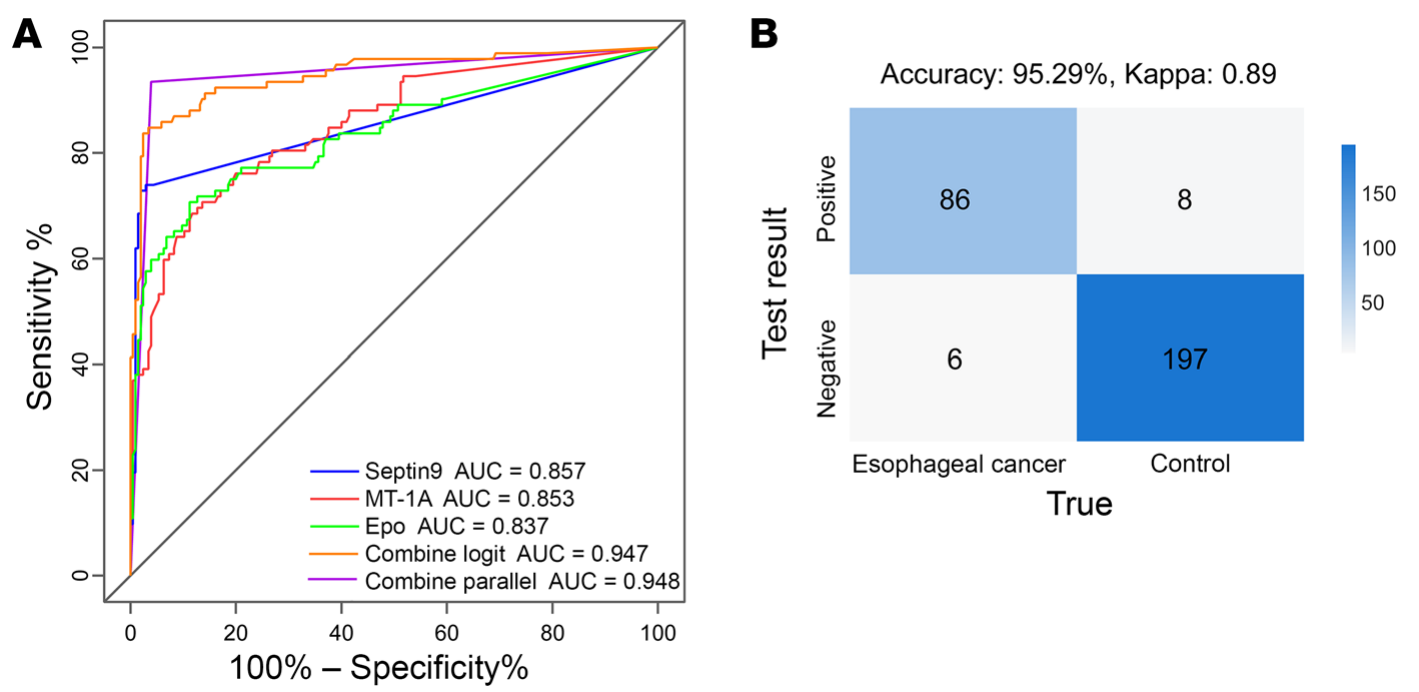
MDM model detection of methylation status by qMSP in the model verification cohort. (**A**) Diagnostic efficacy of the 3 candidate MDMs and the combined panel in samples from the model verification cohort. The ROC curves indicated the performance for distinguishing esophageal cancer (*n* = 92, including 87 patients with esophageal cancer and 5 patients with high-grade intraepithelial neoplasia) from nonesophageal cancer (*n* = 205, including 16 patients with other cancers and 189 healthy individuals). (**B**) Confusion matrix comparing observations we confirmed to be true (reference detection methods) with 3-MDM panel-predicted diagnoses in the model verification cohort. The esophageal cancer (*n* = 92) group, according to observations we confirmed to be true, included 87 patients with esophageal cancer and 5 patients with high-grade intraepithelial neoplasia. The control group (*n* = 205), according to true-observed classifications, included 16 patients with other cancers and 189 healthy individuals.

Using logistic regression and parallel techniques, the combination of candidate MDMs and the diagnostic performance of the model verification cohort were evaluated. The AUC values for the individual ROC curves of Septin9, Epo, and MT-1A were 0.857, 0.853, and 0.837, respectively. When combined, they reached AUC values of 0.947 (logistic regression) and 0.948 (parallel techniques), indicating improved diagnostic accuracy (Figure 3A and Supplemental Table 2). In the integration of 3 MDMs, the AUC values of logistic regression and parallel techniques were similar, with slightly higher AUC for the parallel technique. Therefore, we opted for the parallel technique in our combined approach. Comparison of predicted (by qMSP and parallel techniques) versus observed classifications by confusion matrix showed that this panel of candidate MDMs provided 95.29% accuracy in discriminating between patients with EC and healthy individuals acting as controls, suggesting relatively high consistency between the model and actual clinical diagnoses (Figure 3B, κ = 0.89).

### Clinical validation

#### Demographics in the clinical cohort.

The clinical validation cohort consisted of 641 participants with EC, while the control group comprised 788 participants without EC, including healthy individuals acting as controls and participants with benign esophageal diseases or other types of cancer. Participants with EC were categorized according to the American Joint Committee on Cancer (AJCC) staging system, with 32 participants at stage 0, 106 participants at stage I, 111 participants at stage II, 204 participants at stage III, 117 participants at stage IV, and 71 participants with unknown staging information (Table 1).

To assess the performance of diagnostic marker in confirmed participants with EC and illustrate its applicability for diagnosing EC in high-risk individuals, participants were assigned to the diagnosed group (this group included participants diagnosed with EC and healthy individuals acting as controls before methylation testing, *n* = 534) and the diagnosing group (this group included participants diagnosed with EC and benign esophageal diseases as well as healthy individuals acting as controls after methylation testing, *n* = 697). There were 12 participants at stage 0, 48 participants at stage I, 40 participants at stage II, 88 participants at stage III, 32 participants at stage IV, and 37 participants with unknown staging information in the diagnosed group and 20 participants at stage 0, 58 participants at stage I, 71 participants at stage II, 116 participants at stage III, 85 participants at stage IV, and 34 participants with unknown staging information in the diagnosing group. Besides, the other cancers group comprises 198 cases (age 60.39 ± 10.2 years, 130 male and 68 female), and the distributions of cancer types are as follows: lung cancer accounted for 14.14%, with 28 cases; liver cancer represented 9.6%, with 19 cases; colorectal cancer accounted for 25.76%, with 51 cases; breast cancer accounted for 15.15%, with 30 cases; and gastric cancer was the most prevalent at 35.35%, with 70 cases.

#### ROC analysis.

**Figure 4:**
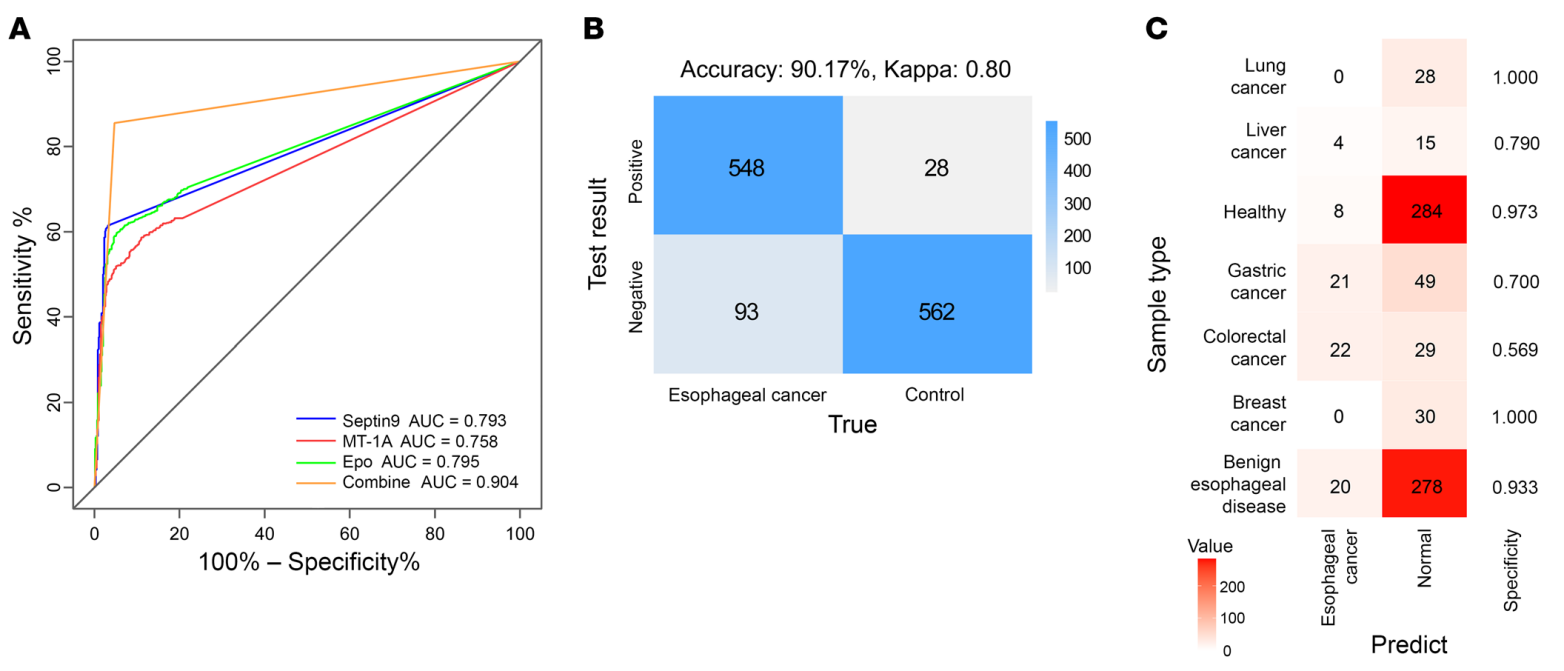
Multiplex detection of Septin9, Epo, and MT-1A methylation status by qMSP in the clinical validation cohorts. (**A**) Diagnostic efficacy of the MDMs and the combined panel in the clinical validation cohort. The ROC curves indicated the performance for distinguishing esophageal cancer (*n* = 641, including 609 patients with esophageal cancer and 32 patients with high-grade intraepithelial neoplasia) from nonesophageal cancer (*n* = 788, including 198 participants with other cancers, 292 healthy participants, and 298 participants with benign esophageal diseases). (**B**) Confusion matrix comparing true-observed classifications (reference detection methods) with 3-MDM panel-predicted diagnoses in the clinical validation cohort. The esophageal cancer (*n* = 641) group, according to true-observed classifications, included 609 patients with esophageal cancer and 32 patients with high-grade intraepithelial neoplasia. The control cases (*n* = 590), according to true-observed classifications, included 298 patients with benign esophageal diseases and 292 healthy individuals. (**C**) Specificities of the 3-MDM panel in each cancer type of 198 participants with other cancers, in 292 healthy participants, and in 298 participants with benign esophageal diseases. Different sample types are listed along the vertical axis, and predictive results are shown in the heatmap, while the corresponding specificity for each sample type is shown on the right vertical axis.

In the clinical validation phase, the MDMs were again validated by qMSP in plasma samples from 609 participants with EC, 32 participants with high-grade intraepithelial neoplasia (EC at stage 0), 298 participants with benign esophageal diseases, 198 participants with other types of cancer, and 292 healthy participants. The original data from qMSP assays were presented in Supplemental Table 3. We assessed the accuracy of the 3 MDMs by ROC analysis in the clinical cohort (*n* = 641 EC samples; *n* = 788 samples without EC). Among the 1,429 clinical samples, ROC curve analysis of qMSP Ct values in individual or multiplex detection assays of Septin9, Epo, and MT-1A yielded AUC values of 0.793, 0.758, and 0.795, respectively, and 0.904 for all 3 markers together (Figure 4A). These results suggested that multiplex detection using these candidates could provide higher diagnostic accuracy compared with detection of any individual MDM. Further comparison by confusion matrix of classifications predicted by qMSP assays of the 3-MDM panel with the observed clinical diagnoses showed an accuracy value of 90.17% (Figure 4B, κ = 0.80).

#### Sensitivity.

To further assess whether the diagnostic efficacy of the 3-MDM panel differed among stages of EC, we compared its sensitivity among participants in the clinical cohort stratified by disease stage. Multiplex qMSP analysis of Septin9, Epo, and MT-1A showed detection sensitivities of 85.49% (95% CI, 82.55%~88.01%) for overall (*n* = 641) patients, and the detection performance showed positive correlation with the tumor progression. The sensitivity of stage 0 (*n* = 32), stage I (*n* = 106), stage II (*n* = 111), stage III (*n* = 204), and stage IV (*n* = 117) was 56.25% (95% CI, 39.06%~73.44%), 77.36% (95% CI, 69.39%~85.32%), 86.69% (95% CI, 80.13%~92.85%), 89.70% (95% CI, 85.54% ~93.88%), and 94.02% (95% CI, 88.06%~97.56%), respectively (Supplemental Figure 4). The sensitivity in different age groups showed no significant difference in the range of 40 to over 80 years (Supplemental Table 4). The sensitivity performance of MDMs was analyzed in tumors with different degrees of differentiation. The medium-high differentiation group showed the highest performance at 92.31% (95% CI, 66.69%~98.63%) (Supplemental Table 5).

#### Specificity.

In assays testing whether our multiplex qMSP method could distinguish individuals acting as controls, including both healthy individuals and patients with benign esophageal disease, the 3-MDM panel achieved a specificity 95.25% (93.21%~96.82%). More specifically, healthy individuals could be identified with 97.26% specificity (95% CI, 94.67%–98.81%), while benign esophageal diseases were diagnosed with 93.29% specificity (95% CI, 89.82%–95.85%). We assembled a cohort of heterogeneous cancer types, including liver, colorectal, breast, and lung cancers, to examine the specificity of these 3 candidate markers for discriminating EC from other cancer types. Specificity decreased to 56.86% (95% CI, 43.27%–70.46%) among patients with colorectal cancer. By contrast, the specificity for detecting lung cancer reached 100.00% (95% CI, 87.94%–100.00%); specificities of 100.00% for breast cancer (95% CI, 88.43%–100.00%) and 78.95% for liver cancer (95% CI, 60.62%–97.28%) were found; and a specificity of 70.00% was found for discriminating gastric cancer (95% CI, 59.26%–80.74%) (Figure 4C and Supplemental Figure 5).

#### Positive predictive value and negative predictive value.

The positive predictive value (PPV) in the overall clinical cohort was 95.14% (548 of 576), while the negative predictive value (NPV) was 85.80% (562 of 655); Septin9 alone showed the highest PPV of 96.72%, and the best single marker NPV was detected by Epo with 65.15% (Table 2).

#### Comparison with conventional tumor markers.

**Figure 5:**
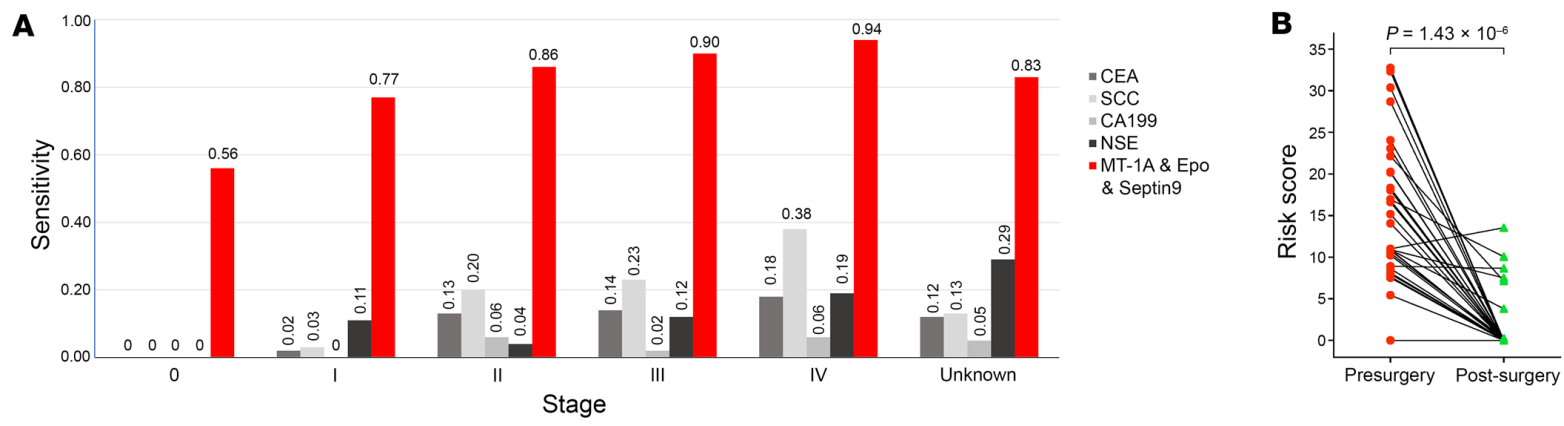
Comparison of the 3-MDM panel with conventional markers and their application in treatment monitoring. (**A**) Comparison of sensitivity between the 3-MDM panel detection method and conventional tumor protein markers in different cancer stages of the clinical validation cohort. The total number of esophageal cancer samples was 609, with the sample sizes for each stage listed in Table 1. (**B**) Preoperative and postoperative methylation levels in a subset of patients (*n* = 32) who underwent complete surgical resection. Wilcoxon’s test was used for pairwise comparison.

Further comparison of the Septin9, Epo, and MT-1A MDM panel with conventional tumor markers in diagnosing different tumor stages in the clinical cohort showed positive detection rates of 56.25%, 77.36%, 86.49%, 89.71%, and 94.02% for cancer stages 0–IV, respectively, which were notably higher than those of the conventional tumor markers CEA, SCC, CA199, and NSE (Figure 5A and Supplemental Figure 6). In addition, we also calculated the Youden index of each tumor marker, and the results showed that the 3-MDM panel detection method was optimal. The respective Youden indexes of CEA, SCC, CA199, NSE, and the 3-MDM panel were 0.11, 0.22, 0.03, –0.02, and 0.76. Furthermore, the sensitivity of the 3-MDM panel for squamous cell carcinoma, adenocarcinoma and other rare cancers showed no significant difference and can be applied to all types of ECs.

#### Performance for high-risk populations in EC.

To further evaluate whether the EC methylation detection method in this study has the potential to be used for screening or as an adjunct diagnostic tool in populations with high-risk for EC, we analyzed performance of the 3-marker panel in the diagnosed group (*n* = 534) and the diagnosing group (*n* = 697), as detailed in Table 1. The sensitivity, specificity, PPV, and NPV in the diagnosed group were 85.21% (80.27%~89.31%), 97.11% (94.39%~98.74%), 96.48% (93.17%~98.47%), and 87.62% (83.41%~91.09%), respectively, compared with 85.68% (81.77%~89.02%), 93.61% (90.30%~96.05%), 94.27% (91.29% ~ 96.46%), and 84.20% (79.93%~87.87%) in the diagnosing group (Table 3). The diagnosing group included populations with high-risk for EC, while the diagnosed group includes participants with confirmed EC as control for the diagnosing group. There is no statistically difference between the diagnosed group and the diagnosing group in terms of sensitivity, specificity, PPV, and NPV, which indicates that the 3-marker panel can be used for screening or diagnosing populations with high-risk for EC.

#### Treatment monitoring.

Additionally, examination of postoperative methylation levels in a subset of participants who underwent complete surgical resection revealed that 29 of 32 (90.6%) participants tested negative for methylation in the Septin9, Epo, and MT-1A promoter regions on the third day after surgery. The methylation risk scores (45-ΔCT) of most participants included in treatment monitoring decreased after surgery compared with those before surgery, showing statistical significance (Wilcoxon’s test, *P* < 0.001) (Figure 5B).

## Discussion

This study introduces a noninvasive approach for the detection of EC using gene methylation profiles in plasma samples, offering marked advantages over the conventional invasive endoscopic and pathological examinations that are often painful and less accessible and thus impede early diagnosis and treatment. By screening EC methylation chip data in public databases, along with internal plasma WGBS data, including EC at an early stage, 31 differentially methylated regions were identified. Subsequent logistic regression analysis of 31 differentially methylated regions in esophageal and nonesophageal cancer samples pinpointed 6 differentially methylated regions with a strong association with EC. Subsequently, they were verified using qMSP technology in cancer cell lines and clinical plasma samples, leading to the selection of 3 genes — MT-1A, Epo, and Septin9 — for the development of a methylation-based detection method for EC.

In our study, preclinical plasma sample verification was conducted, followed by a case-control and multicenter clinical study with sufficient participants to validate the effectiveness of this method. The results indicated high consistency with clinical gold standard, with superior sensitivity and specificity compared with existing studies and commonly used tumor markers, particularly for early-stage EC at stage 0 and I. The approach of combining the detection of MT-1A, Epo, and Septin9 gene methylation for EC diagnosis, which we believe was introduced by this study, is a strategy not previously documented to our knowledge. The combining method was more effective in the diagnosing EC compared with single gene methylation detection.

From the perspective of clinical study design, this research utilized a multicenter trial approach, which enables the inclusion of a larger number of participants within the same time frame compared with a single-center trial, thereby reducing the duration of the clinical trial. Multicenter trials involve collaboration among various regions, different trial institutions and numerous clinical researchers, leading to conclusions that are often broadly representative.

In this clinical validation stage, we employed a strategically designed 2-group cohort to thoroughly evaluate our biomarkers’ diagnostic performance. The diagnosed group included patients who had already been diagnosed with EC at the time of methylation testing. By comparing the methylation detection results with confirmed diagnoses in this group, we could robustly assess the accuracy and reliability of the DNA methylation markers. The diagnosing group comprised high-risk individuals who had not yet been definitively diagnosed with EC at the time of methylation testing. After performing the methylation testing on this group, the diagnosis was definitively made to simulate applications of methylation testing in real clinical situation. We later correlated the results of methylation testing with the definitive diagnostic outcomes and conducted integrated analysis. This setup allowed us to examine the practical application of the DNA methylation testing in the assessment of individuals at high-risk for EC. Remarkably, the performance characteristics observed in the diagnosing group were consistent with those in the diagnosed group, demonstrating a promising tool for early detection in high-risk populations. Therefore, this 2-group design provides compelling evidence that our biomarkers can be effectively utilized for both clinical diagnosis and early screening of EC in high-risk individuals, highlighting the broad diagnostic potential.

The metallothionein (MT) family is a low-molecular-weight protein family known for its strong affinity toward metal ions (9). This protein family consists of isomers and plays a crucial role in regulating the homeostasis and oxidation of transition metal ions with cells. Among its various functions are the maintenance of cellular balance as well as involvement in processes such as cell proliferation, differentiation, and apoptosis. MT-1A is 1 of the 4 isoforms in MT family, and aberrant MT expression has been observed in several human tumors, including EC, gallbladder cancer, B cell lymphoma, breast cancer, liver cancer, skin cancer, papillary thyroid cancer, and prostate cancer (10). Studies have demonstrated that overexpression of MT can shield cancer cells from free DNA damage and lipid peroxidation induced by free radicals (11). Recent investigations have highlighted elevated expression of MT in squamous cell carcinoma, suggesting its potential utility as a diagnostic marker for ESCC (12). Erythropoietin (Epo) is a glycoprotein hormone (13). Chan et al. discovered that Epo can rapidly induce the expression of the proto-oncogene c-myc, exert antiapoptotic effects, and promote cell survival (14). Septin is a conserved family of skeleton protein genes with GTPase activity found in all eukaryotes except plants and it plays a role in cell division. In humans, the family comprises 14 members designated as SEPT 1–14. Research has indicated a direct association between Septin9 and tumor development, with varying expression and function across different tumor types (15). Particularly, Septin9 is highly expressed in gastrointestinal tumors (16, 17), serving as a reliable marker for their detection.

Numerous studies have explored gene methylation markers for EC. For instance, Qin et al. used quantitative allele-specific real-time target and signal amplification technology to develop a diagnostic model based on 5 methylation genes, achieving a specificity of 91%, detecting 74% of 84 ECs, with a sensitivity of 43% for 14 stage I cancer and no cancer at stage 0 (8). Although they used a similar framework, our study employed WGBS and included both tissue and plasma samples from a larger cohort in the marker discovery stage. These methodological differences in data coverage, sample types, and sample sizes led to distinct gene signatures, emphasizing the robustness and specificity of our approach in identifying reliable biomarkers (8). Li et al. established a diagnostic methylation classifier based on 12 CpG sites, effectively distinguishing BE, EAC, and ESCC from normal tissues (AUC = 0.992) (18). However, this study was solely based on bioinformatics analysis without validation using clinical plasma samples. Salta et al. utilized qMSP to assess the efficacy of detecting EC tissue using 2 methylated gene combinations (19). Their study achieved the identification accuracy of 82.29% for adenocarcinoma and 81.73% for squamous cell carcinoma tissue, which was lower than that shown in our study. Qiao et al. employed targeted methylation sequencing technology and a support vector machine algorithm to develop an early detection classifier for EC based on 921 differentially methylated regions by sophisticated deep-targeted sequencing, with a sensitivity of 74.7% and a specificity of 95.9% in 181 clinical samples (20). The sensitivity for detecting stage 0–II EC was lower than that observed in the current research.

Conventional tumor markers commonly used for adjunctive diagnostic, prognosis, and therapeutic monitoring purposes in EC include cytokeratin-21-1-fragment (CYFRA21-1), carcinoembryonic antigen (CEA), squamous epithelial cell carcinoma antigen (squamous cell carcinoma antigen [SCC]), and tissue polypeptide-specific antigen (TPS), etc. While combined application of these tumor markers may enhance efficiency in the intermediate and advanced stages of EC, the individual sensitivity of them for EC at an early stage is generally below 20%. Our study not only confirmed the high diagnostic accuracy of the methylation-based approach but also demonstrates its superiority over conventional tumor markers like CYFRA21-1, CEA, SCC, and TPS. Furthermore, there was a notable enhancement in specificity among individuals exhibiting symptoms of EC but not gastrointestinal cancer.

In the study, there were 534 individuals in the diagnosed group and 697 individuals in the diagnosing group. The sensitivity results observed were consistent, suggesting that the screening method is suitable for identifying EC in suspected and high-risk populations. The accuracy of the EC methylation detection method in the model verification cohort (95.29%) was higher than that in the clinical validation cohort (90.17%), possibly due to differences in sample size, sample heterogeneity, and experimental errors. The findings from methylation testing for pre- and postsurgery patients indicated that 90.6% of patients exhibited a negative methylation status following the surgical procedure, leading to a notable reduction in overall methylation levels. However, when we compared the methylation risk scores of patients with EC before and after surgery, we observed that 3 patients did not experience a decline after treatment. For one patient, the preoperative methylation levels of the biomarkers were already very low, resulting in a false-negative diagnosis before surgery. This might be attributed to individual differences, as some patients with EC do not exhibit abnormal methylation in peripheral blood cell-free DNA (cfDNA). Consequently, this patient’s postoperative methylation risk score did not decrease. For the other 2 patients, the exact mechanisms underlying the lack of a decline in methylation risk scores remain unclear. In future studies, we intend to conduct a dedicated investigation to evaluate the performance of this EC biomarker in treatment monitoring and better understand the clinical implications of changes in methylation risk scores during the treatment process.

### Conclusions.

In conclusion, a robust association exists between the development of EC and integrated test of MT-1A, Epo, and Septin9 methylation. We offer a promising, highly accurate method not only for the early detection of EC and individuals with high-risk for EC, but also for the therapeutic monitoring.

## Methods

### Sex as a biological variable

Our study examined male and female humans, but sex was not considered as a biological variable.

### Study design and patient cohorts

Two cohorts of participants are enrolled in this study (the model-verification cohort containing 297 participants and the clinical validation cohort containing 1,429 participants). The participants were prospectively recruited, including participants with EC, participants with benign lesions, healthy individuals acting as controls, and participants with other cancers, from multiple centers in China.

The inclusion criteria are as follows below.

#### EC.

The included participants were those over 40 years old who satisfied any of the following criteria: participants with long-term residence in areas with high EC incidence or with family disease history, participants with symptoms of upper gastrointestinal discomfort, participants with the presence of precancerous lesions of EC, participants with a strong clinical suspicion of EC or high-grade intraepithelial neoplasia based on endoscopic, imaging, or pathological biopsy findings; and participants with benign digestive system diseases who intend to receive an endoscopy test or with prior endoscopic findings. All the included participants in this classification have no history of EC surgery and no prior treatment.

To validate the diagnostic performance of the MDMs in EC patients, while also to demonstrate its potential application in populations with high risk of EC, we divided the included patients of the clinical validation cohort into a diagnosed group and a diagnosing group. In the diagnosed group, the participants were diagnosed definitively first and then provided blood for subsequent methylation testing. In the diagnosing group, the participants provide blood for methylation testing first and then started definitive diagnosing procedures to simulate applications of methylation testing in real clinical situation. EC was diagnosed based on characteristics observed during upper gastrointestinal endoscopy, computed tomography, or magnetic resonance imaging and confirmed through histopathology. Tumor staging was determined according to the AJCC/Union for International Cancer Control 8th edition. During all the double-blinded experimental processes, participant information was kept confidential from experimental operators and researchers to ensure the credibility and reliability of clinical trial outcomes.

#### Benign esophageal conditions.

Participants were clinically diagnosed with other esophageal diseases (such as reflux esophagitis, achalasia, esophageal hiatal hernia, diffuse esophageal spasm, and irregular esophageal spasm) based on laboratory tests (tumor markers, bronchoscopy, or imaging, etc.), with no evidence of EC, requiring further evaluation and management.

#### Other types of cancer.

Participants who had not undergone treatment or surgery were clinically diagnosed with other cancers, such as gastric cancer, colorectal cancer, etc.

#### Healthy individuals acting as controls.

Participants with no history of malignant tumors, who were clinically confirmed to be free of EC other digestive diseases, and those with substantial medical conditions such as hepatitis, cirrhosis, and chronic obstructive pulmonary disease were enrolled as control participants. All healthy volunteers underwent a series of routine health assessments, including complete blood counts, urinalysis, blood biochemistry tests, electrocardiograms, low-dose chest computed tomography, and abdominal ultrasound examinations.

### Sample collection and preparation

The blood samples from patients with EC and other groups were collected from multiple hospitals in China, including Cancer Hospital of the Chinese Academy of Medical Sciences, The First Affiliated Hospital of the Air Force Medical University, Henan Provincial Cancer Hospital, The Second Affiliated Hospital of the Air Force Medical University, The Second Affiliated Hospital of Xi’an Jiaotong University, and The Affiliated Cancer Hospital of Shantou University Medical College. Blood samples collected from all participating hospitals were processed following the same protocol by trained technicians. A 5 mL K_2_EDTA anticoagulant tube (BD Vacutainer) was used to collect a 5 mL peripheral blood sample to ensure the accuracy of the tests. Samples were processed and transported following the guidelines for nucleic acid extraction reagent (BioChain [Beijing] Science & Technology Inc.) Plasma was separated from whole blood by centrifugation within 4 hours of blood sample collection and stored immediately at −80°C. Plasma was tested within 2 weeks of collection.

### DNA extraction and bisulfite conversion

DNA extraction and bisulfite conversion were carried out following the instructions provided in the manufacturer’s manual for the nucleic acid extraction reagent (BioChain [Beijing] Science & Technology Inc.).

### qMSP

When designing primers and probes for qMSP, primers were designed to include at least 1 CpG site in both forward and reverse primers, as well as in the probe binding sequence, ensuring that only methylated DNA templates were amplified. We extract cfDNA from cell-free plasma to prepare DNA templates. We simultaneously detect a reference gene, ACTB, when testing target genes. Through extensive clinical sample validation, we established that cfDNA content in plasma should not fall below 0.9 ng/mL. Consequently, we set a reference ACTB Ct value threshold of ≤34.8. If the reference gene met this criterion, the sample was deemed suitable for analysis, allowing us to determine the presence or absence of methylation in the marker gene. The sulfite-modified DNA served as the template for qMSP, following the detailed procedures outlined in the MT-1A, Epo, and Septin9 methylated gene detection kit (BioChain [Beijing] Science & Technology Inc.), which employs the PCR fluorescent probe method. The amplification reactions were conducted in a total volume of 50 μL, consisting of 25 μL reaction buffer and 25 μL sulfite-modified DNA template. The amplification process was conducted using either the Applied Biosystems 7500 Fast Real-Time PCR System or the SLAN-96S Fully Automatic Medical PCR Analysis System. Each experimental batch included patient DNA samples and positive controls and negative controls to maintain stringent quality control throughout the analysis.

### Marker discovery

In this study, a total of 108 EC cancer tissue samples, 107 adjacent normal tissue samples, and 249 healthy human WBC (white blood cell) samples from public datasets were analyzed. To integrate data from two methylation detection chips, a mapping and matching process was conducted for the detection probes based on specific criteria. These criteria included ensuring that the probe design intervals overlapped in the genome coordinates or that the maximum distance between probes did not exceed 150 base pairs. Additionally, probes from the Human Methylation 450 chip (Illumina, Inc.) and the GoldenGate chip (Illumina, Inc.). Cancer-specific hypermethylation markers were identified based on the following criteria: adjusted *P* < 1 × 10^–2^, delta_T2N (difference in methylation levels between tumor and adjacent normal tissues) >0.1 and mean_wbc (mean methylation level in WBCs) <0.1.

The public data sets utilized in this research, including GSE51287, GSE26784, GSE40279, GSE52826, and GSE74693, were obtained from the NCBI GEO database (https://www.ncbi.nlm.nih.gov/gds). These datasets exclusively consisted of methylated chip data based on two kinds of platforms, the Human Methylation 450 chip and the GoldenGate chip. Notably, data in GSE40279 specifically included samples from individuals aged between 30 and 60 years.

Furthermore, a subset of the in-house plasma samples were utilized as a discovery-step validation set to confirm the markers previously selected based on the public datasets mentioned previously. The in-house samples were from 107 healthy individuals and 56 patients with EC at early stage. These samples underwent WGBS assay. Given the presence of strong background signals in plasma detection outcomes, a one-hot approach was utilized to delineate the identification of cancer-specific hypermethylation signal patterns in plasma. It is a method for categorizing methylation signals based on a predefined threshold. For each methylation DNA region, if the methylation level in a sample exceeds the detection threshold, it is classified as “detected,” with the signal value set to the methylation level; otherwise, it is classified as “not detected,” with a signal value of 0. The threshold was set at the 95th percentile of plasma methylation levels in individuals without cancer to achieve 95% specificity in these control participants, minimizing false positives and allowing for the identification of suitable candidate markers. During this screening process, a total of 31 marker intervals were examined.

We utilized the methylation levels of EC differential methylation regions and the sample type (EC vs. nonesophageal cancer) to establish a logistic regression diagnostic model for EC. The variables included in the model will be used as highly relevant candidate biomarkers for EC for subsequent validation through qMSP analysis.

For the qMSP analysis, we used DNA from HeLa cells (75 ng), which was verified by Sanger sequencing to be highly methylated for Epo, MT-1A, PDGFRA, HOXB13, TRIM15, and Septin9, as the positive control, and WBC DNA (35 ng) as the negative control. Clinical blood samples from patients with EC (*n* = 20), benign esophageal diseases (*n* = 10), and healthy individuals (*n* = 12) were also tested. This screening process effectively validated the methylation biomarkers related to EC.

### Model verification

To evaluate the diagnostic efficacy of the methylation-based markers for EC, a model-verification cohort comprising various participant groups was assembled, including patients diagnosed with EC, individuals with benign esophageal conditions, patients with other types of cancer, and healthy individuals. The model-verification cohort consisted of 87 patients with EC, 5 patients with high-grade intraepithelial neoplasia, 16 patients with other cancers, 189 healthy individuals. The blood samples were collected from individuals of this cohort to perform qMSP. The experimental details were described above.

### Clinical validation

A multicenter, parallel comparison, blinded clinical trial design was utilized, with inclusion criteria consistent described above. The samples were used to assess the diagnostic efficacy of EC methylation gene detection technology.

The clinical trial was approved prior to the commencement of the study. This clinical validation cohort consisted of 609 patients with EC, 32 patients with high-grade intraepithelial neoplasia, 298 patients with benign esophageal disease, 198 patients with other cancers, and 292 healthy individuals. The blood samples were collected from individuals of this cohort to perform qMSP. The experimental details were described above.

The prespecified primary outcomes of this clinical trial were sensitivity and specificity, which are the key measures of diagnostic performance for the biomarkers being evaluated. The analyses presented in the manuscript focus on the prespecified primary outcomes (sensitivity and specificity).

### Clinical trial information

The number for this clinical trial is ChiCTR2400083525.

### Medical device registration information

The National Medical Products Administration registration number is 20243401368.

### Statistics

Descriptive analysis of the demographic characteristics and initial participant data were conducted. Categorical variables were summarized using frequency and percentage composition, while quantitative variables were summarized using measures such as mean, standard deviation, and median. The diagnostic efficacy was assessed through diagnostic test evaluation, including comparison with the gold standard and calculation of κ values and their corresponding 95% CI (21). Sensitivity was defined as the proportion of correctly identified positive EC cases among all EC cases, while specificity was defined as the proportion of correctly identified negative cases among all normal/esophageal benign disease and other cancer cases. PPV and NPV were calculated to determine the probability of a positive or negative disease test result, respectively. Receiver operating characteristics (ROC) curves were generated using R software, and the area under the ROC curve (AUC) was analyzed. For sample sizes lower than 5, the association between test positivity and demographic characteristics was assessed using either the χ^2^ test or Fisher’s exact test. Statistical significance was defined as *P* < 0.05.

### Study approval

Prior to sample collection, all participants provided written consent and were duly informed about the utilization of sample and test outcomes. Approval for this study was obtained in 2021 from the medical ethics committees of the hospitals that participated in the study: Cancer Hospital of the Chinese Academy of Medical Sciences (21/223-2894); The First Affiliated Hospital of the Air Force Medical University (Xi’an, China) (QX20211043-x-1); The Second Affiliated Hospital of the Air Force Medical University (Xi’an, China; 202110-09); Henan Provincial Cancer Hospital (Henan, China; 2021-240B-001); The Second Affiliated Hospital of Xi’an Jiaotong University (2021 ethical 056); and The Affiliated Cancer Hospital of Shantou University Medical College (2021019). The recruitment of participants and sample collection were conducted after obtaining ethical approvals, and the trial was registered with the Chinese Clinical Trial Registry (ChiCTR) in April 2024.

### Data availability

The values associated with the data points shown in graphs and values behind any reported means are presented in the Supporting Data Values file as are the qMSP data and the detailed Ct values in the 3 stages of present study. The WGBS data for the in-house cohort utilized in marker discovery step can be viewed in the Genome Sequence Archive database (accession PRJCA035851; https://ngdc.cncb.ac.cn/bioproject/browse/PRJCA035851).

## Author contributions

RZ, Y Nie, XC, TJ, JW, YP and Guangpeng Zhou are co–first authors of the article. The order of the co-first authors was determined based on their respective contributions to the clinical trial and their roles as representatives of the seven participating research institutions. Specifically, the order reflects the importance of their contributions to the study, such as the number of samples collected, the quality of data provided, and their involvement in key aspects of the research process. RZ is listed first due to the leading role in sample collection and data coordination. These co–first authors conducted the experiments and had full access to all data in the study and take responsibility for the integrity of the data and the accuracy of the data analysis. GJ, XH, JH, and Yin Li were responsible for the study conception and design. Yong Li, LZ, BC, Y Ni, YC, and YX were responsible for managing patients and data acquisition. Analysis and interpretation of the data were conducted by ZZ, XG, ZW, TL, Jie Zhao, CL, Gang Zhao, JC, and Jing Zhao. Drafting the manuscript was performed by RZ, Y Nie, XC, TJ, JW, YP, Guangpeng Zhou, GJ, XH, JH, and Yin Li. RZ, ZZ, ZW, Jie Zhao, XH, and Yin Li checked and confirmed the conclusion and the final manuscript.

## Supplementary Material

Supplemental data

ICMJE disclosure forms

Supplemental tables 1-5

Supporting data values

## Figures and Tables

**Table 1:**
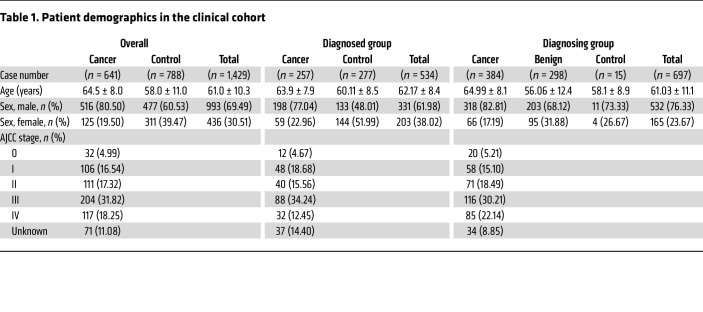
Patient demographics in the clinical cohort

**Table 2:**
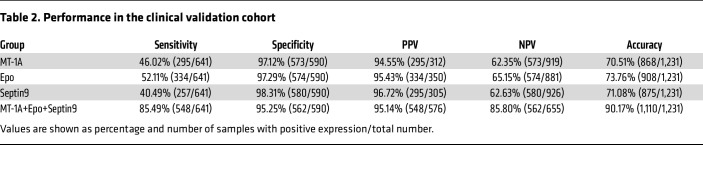
Performance in the clinical validation cohort

**Table 3:**
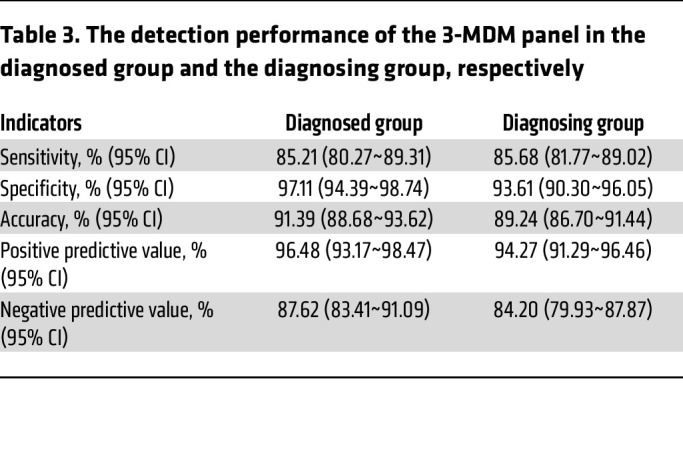
The detection performance of the 3-MDM panel in the diagnosed group and the diagnosing group, respectively

## Version 1 02/25/2025

In-Press Preview

## Version 2 04/15/2025

Electronic publication
