# Supplementary material for: A multicenter prospective clinical trial reveals cell-free DNA methylation markers for early esophageal cancer
Source: J Clin Invest. 2025 Feb 25;135(8):e186816. doi: 10.1172/JCI186816 (PMC11996849; doi:10.1172/JCI186816)
Supplement: Supplemental data [file jci-135-186816-s210.pdf]

Supplementary Figures

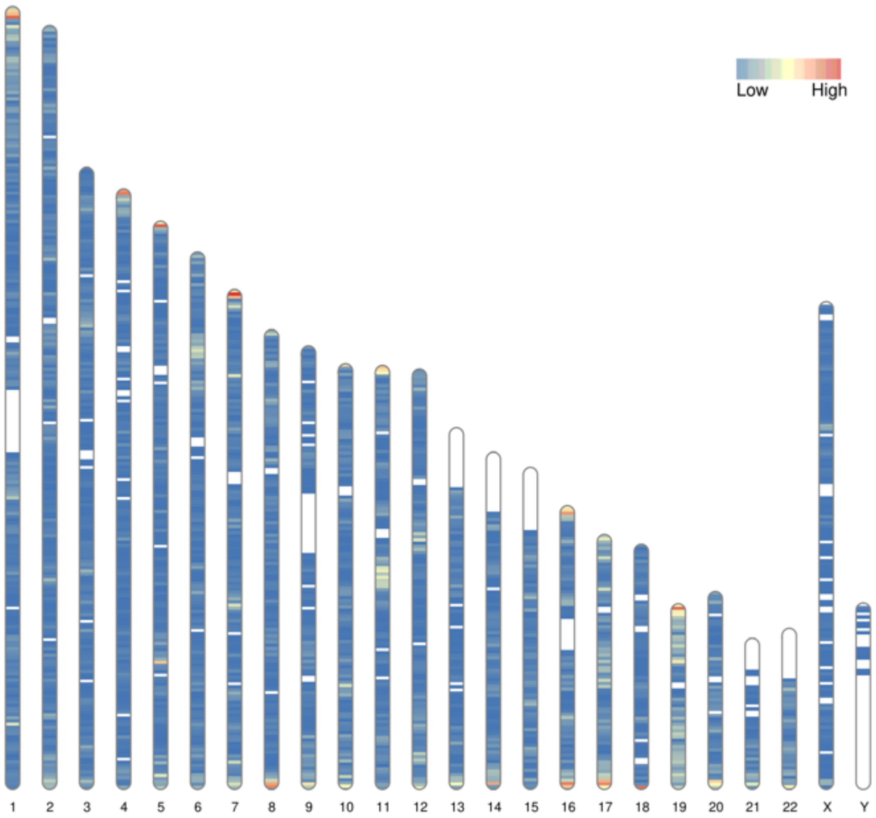

Supplementary Figure 1. Overall methylation level in different chromosomes. The high methylation regions were shown in orange and red.

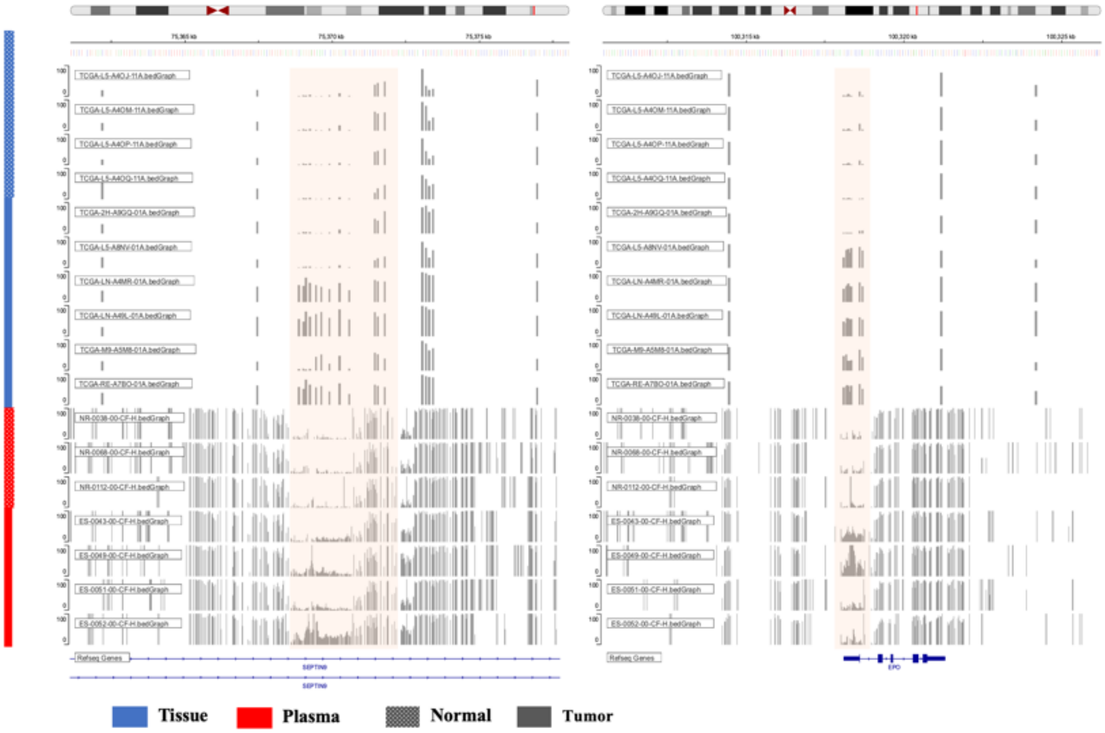

Supplementary Figure 2. Genome browser view of methylation level in selected genes between normal and tumor patients with tissue or plasma samples.

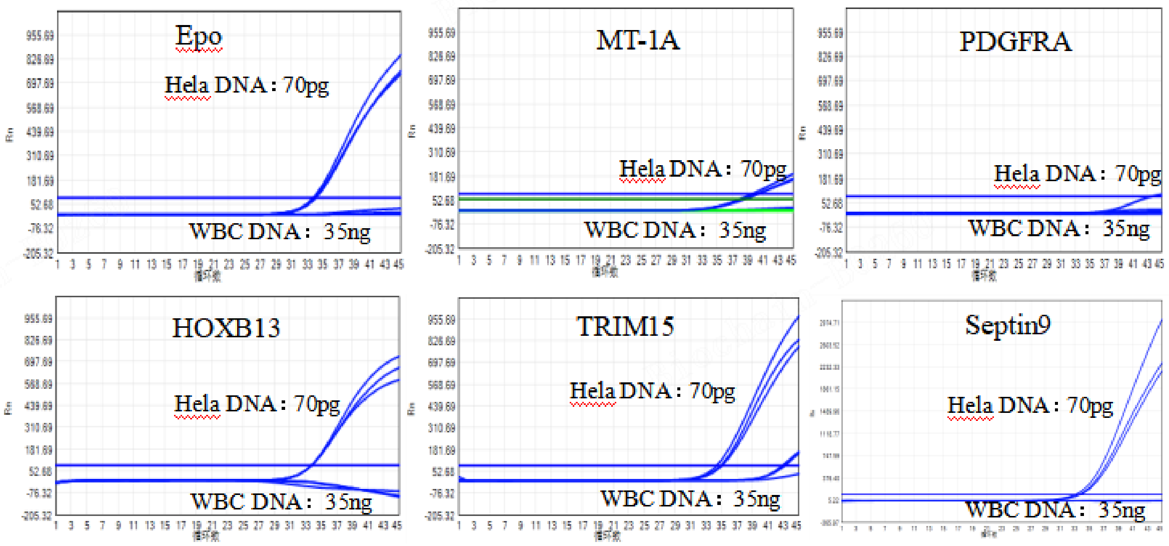

Supplementary Figure 3. Results of qMSP testing for six esophageal cancer differential methylation genes in Hela cells and WBC cell DNA. EPO, MT-1A, HOXB13, TRIM15, and Septin9 showed relatively standard amplification curves and higher signal values in the qMSP testing of Hela cells, while EPO, MT-1A, PDGFRA, HOXB13, and Septin9 genes exhibited no significant amplification curves in the qMSP testing of WBC cell DNA.

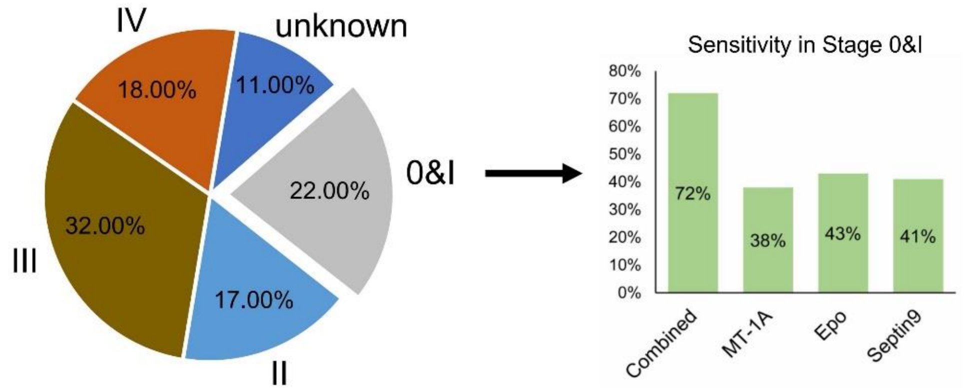

Supplementary Figure 4. The proportion of each stage in clinical validation and the positive rate of MDM panel and combined detection of MDM in early esophageal cancer (stage 0&I).

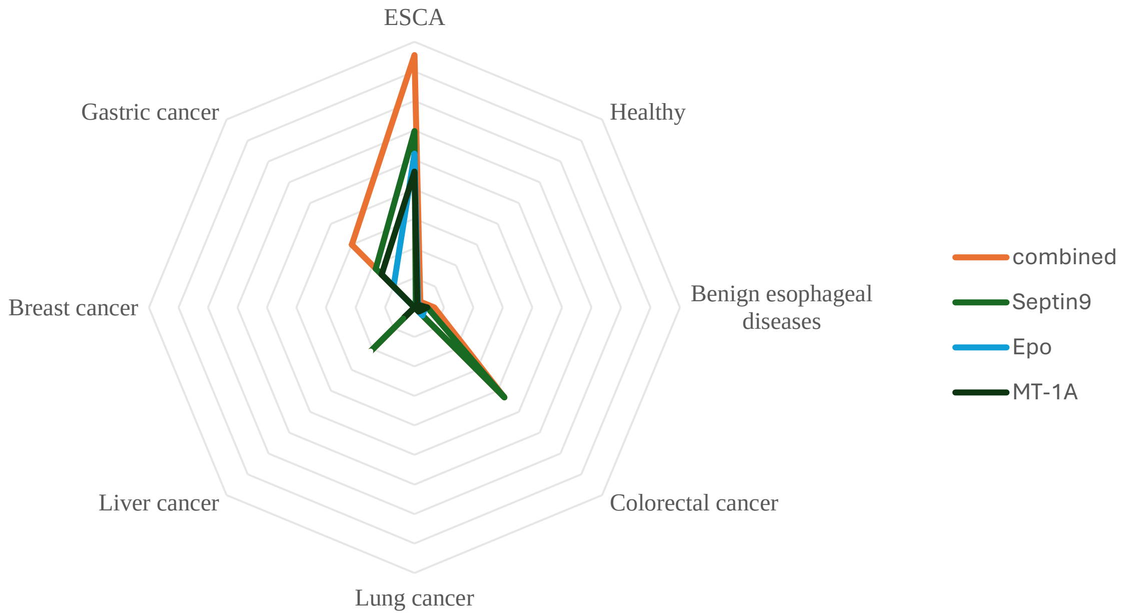

Supplementary Figure 5. The specificity of MDM panel with healthy control, benign esophageal diseases, and other cancer types in radar plot.

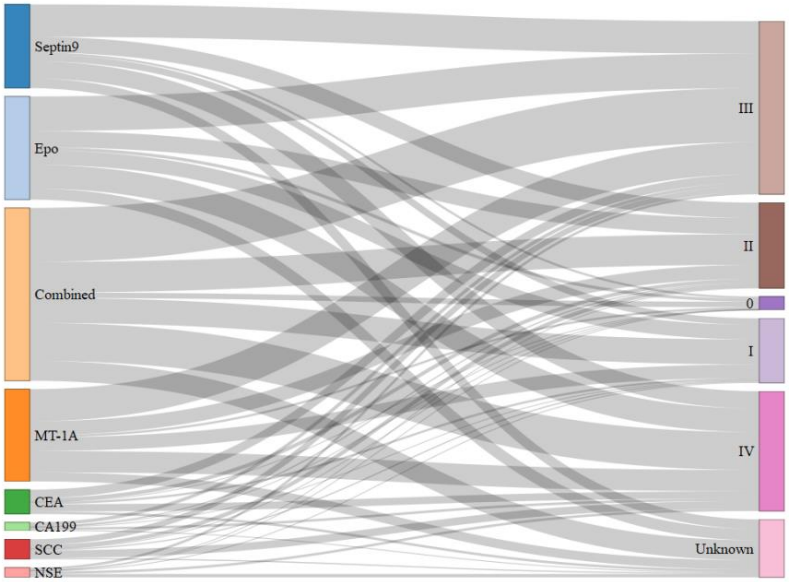

Supplementary Figure 6. The Sankey plot showed comparison of identification EC patients by tumor protein marker (CEA CA199, SCC, NSE) or methylation markers in 0 to IV stages.
